# Large-scale spiking circuit simulation of spatio-temporal dynamics in superior colliculus

**DOI:** 10.1186/1471-2202-15-S1-P4

**Published:** 2014-07-21

**Authors:** Richard E Veale, Tadashi Isa, Masatoshi Yoshida

**Affiliations:** 1 Cognitive Science Program, Indiana University, Bloomington, IN, USA; 2 Dept. of Developmental Physiology, NIPS, Okazaki, Japan; 3 Dept. of Physiological Sciences, Graduate University for Advanced Studies (SOKENDAI), Hayama, Japan

## 

We present computational simulations of the intrinsic circuitry of the superior colliculus using large-scale spiking neural circuit models. We reproduce recent results from slice experiments that showed different spatio-temporal patterns of interaction within the visual layers versus the eye-movement related layers of the superior colliculus. Specifically, the receptive fields of neurons in the visual layers implement a “center-surround” pattern of spatial competition, and furthermore additional input within the central region sums super-linearly. In contrast, the receptive fields of neurons in the motor-related regions implement spatially symmetric fields of overlaid excitation and inhibition, and additional inputs sum linearly. Our simulations investigate the circuit mechanisms and dynamics that differentiate the computational roles of these distinct but related regions.

**Figure 1:**
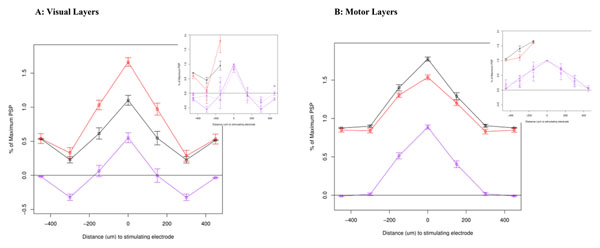


We constructed full-scale simulations of mouse brain slices using spiking neuron models. Connection parameters were then fit within physiological constraints to reproduce experimental data. Figure 1A shows results from a best fit of the superficial (visual) layers. Stimulation was applied at different horizontal distances from a central neuron in a tangential slice. The figure shows integrated post-synaptic potential (PSP) during stimulation at the indicated distance (purple). We also reproduced data from a “two-point” paradigm in which stimulation was simultaneously applied to the indicated electrode and the central electrode, simulating a “large” stimulus (red). The net response of the neuron sums super-linearly in comparison to the linear summation of the results from the two points independently (naïve summation in black). Insets show corresponding slice data. Interestingly, anatomically distinct inhibitory and excitatory populations were not necessary to reproduce spatial asymmetry (“center-surround”) in the visual superficial layers. Rather, asymmetries in the *temporal properties* (synaptic dynamics) of connections were better able to account for observed data under all conditions. A further population of small disinhibitory neurons was necessary to maintain linear summation in the periphery while summing super-linearly in the center.

This contrasts with the motor-related intermediate layers (Figure 1B), whose response to stimulation was best explained by both spatial and temporal symmetry between inhibitory and excitatory neural connections. Overall, the regions seem to have taken advantage of both spatial and temporal dynamics in their connections to specialize their computational function: The visual layers seem geared towards spatial competition and strengthening of salient stimuli, whereas the motor-related regions seem geared towards broad and long-term integration of input.

